# Effects of melatonin on planaria head regeneration are dependent on both timing and duration of exposure

**DOI:** 10.14814/phy2.70151

**Published:** 2025-01-21

**Authors:** Simon C. Beeching, Hanna E. Ruland, Katelyn M. Sparks

**Affiliations:** ^1^ Department of Biology Slippery Rock University of Pennsylvania Slippery Rock Pennsylvania USA

**Keywords:** healing, hormesis, melatonin, morphogen, neoblast, planaria, regeneration

## Abstract

Melatonin is a multifunctional biomolecule with demonstrated stimulatory, inhibitory, and antioxidant effects, including both receptor‐mediated and receptor‐independent mechanisms of action. One of its more perplexing effects is the disruption of regeneration in planaria. Head regeneration in planaria is a remarkable phenomenon in which stem cells (neoblasts) migrate to the wound site, proliferate, then differentiate into all functional tissue types within days of injury. We investigated how both the timing and duration of melatonin exposure affect head regeneration in the planaria *Phagocata gracilis* (Haldeman). Our results demonstrate that *P. gracilis* is capable of recovery from the melatonin‐induced delay of regeneration and reveal the time required to recover to control levels. Further, we found evidence of regenerative stage‐specific responses to discontinuous melatonin exposure, including non‐inhibitory effects. Further exploration of melatonin's effects on regeneration can be targeted to specific regenerative processes, and the possibility of multiple mechanisms of action should be recognized.

## 1 INTRODUCTION

Although best known for its roles as a vertebrate hormone and mitochondrial antioxidant, melatonin's effects on simple tissue repair, regeneration, and therapeutics have received increasing scrutiny among researchers. Studies using mammalian models have revealed melatonin's potential as a promoter of wound healing (Liu et al., 2020), in regenerative medicine (Majidinia et al., 2018), and in facilitating successful organ transplantation (Esteban‐Zubero et al., 2016). These studies ascribe multiple enhancing effects to exogenous melatonin treatment, including stimulating cell proliferation, recruitment of cells, and up‐regulation of cell function. However, inhibitory, apoptotic, and suppressive effects of melatonin treatment during wound healing have also been demonstrated (Bizzarri et al., 2013; Histing et al., 2012; Kobayashi‐Sun et al., 2020). Parallel studies using invertebrate models have yielded similarly confounding conclusions regarding melatonin's effects across an array of both basal and derived metazoans (Anisimov, 2003; Roopin & Levy, 2012).

The apparent contradiction in these assessments of melatonin's pharmacological effects may be due to dose‐dependency of responses, particularly biphasic or “hormetic” responses, where low‐dose stimulation is contrasted by higher‐dosage inhibition or even toxicity (Agathokleous et al., 2019; Calabrese, 2013). Moreover, melatonin exhibits both receptor‐mediated (Jockers et al., 2016) and receptor‐independent (Reiter et al., 2016) effects that can further compound dosage and timing effects. Regeneratively competent animals, such as sponges, cnidarians, amphibians, and turbellarian flatworms (i.e., planaria) provide excellent models for investigation of such complex, pleiotropic effects of morphogens (Fujita et al., 2021; Huizar et al., 2020). Melatonin treatment disrupts normal regeneration in some planaria species (Yermakova et al., 2009; Yoshizawa et al., 1991) and induces dose‐dependent morphogenic and lethal responses in at least one (Beeching & Merritt, 2019). Both timing and duration of exposure to melatonin can determine how exogenous melatonin treatment affects wound healing (Luchetti et al., 2023) but their effects on regeneration are unclear.

In the freshwater planarian *Phagocata gracilis*, continuous exposure to sub‐lethal concentrations of melatonin produces abnormal morphology and a reduced regenerative rate in a dose‐dependent fashion (Beeching & Merritt, 2019). Thus, *P. gracilis* represents an appropriate model for the further exploration of melatonin's effects on regenerative tissues. We compared the effects of continuous melatonin exposure to pulsatile (i.e., episodic) exposure in head‐regenerating *P. gracilis*. Our goal was to first characterize the kinetics of normal and continuous melatonin‐treated head regeneration in *Phagocata gracilis*. Then, we examined timing and stage‐specific differences in melatonin's regenerative effects and explored the hypothesis that head regeneration exhibits critical periods of susceptibility to melatonin.

## 2 MATERIALS AND METHODS

### 2.1 Collection, handling, and treatment

*Phagocata gracilis* were collected throughout the years 2019–2023 from two sites within a single stream located in Slippery Rock, Pennsylvania, USA (80.04 W, 41.07 N) using submerged, baited traps (following Kenk, 1972). We chose *P. gracilis* as it is locally abundant, relatively large (Kenk, 1970), amenable to laboratory regeneration study (Beeching & Merritt, 2019) and not known to undergo spontaneous fission (Hyman, 1937). Following capture, subjects were maintained between 18 and 23°C in the laboratory. Subjects were housed in 38 L all‐glass aquaria with continuously filtered and aerated dechlorinated tap water, with flat rocks providing refuge. Feeding was performed every 2 weeks and consisted of freshly caught and macerated earthworms (Annelida; Oligochaetae). Only subjects that were fasted at least one full week were used experimentally. Flexible plastic pipettes were used to transfer subjects when necessary.

### 2.2 Decapitation, randomization, and scoring

**FIGURE 1:**
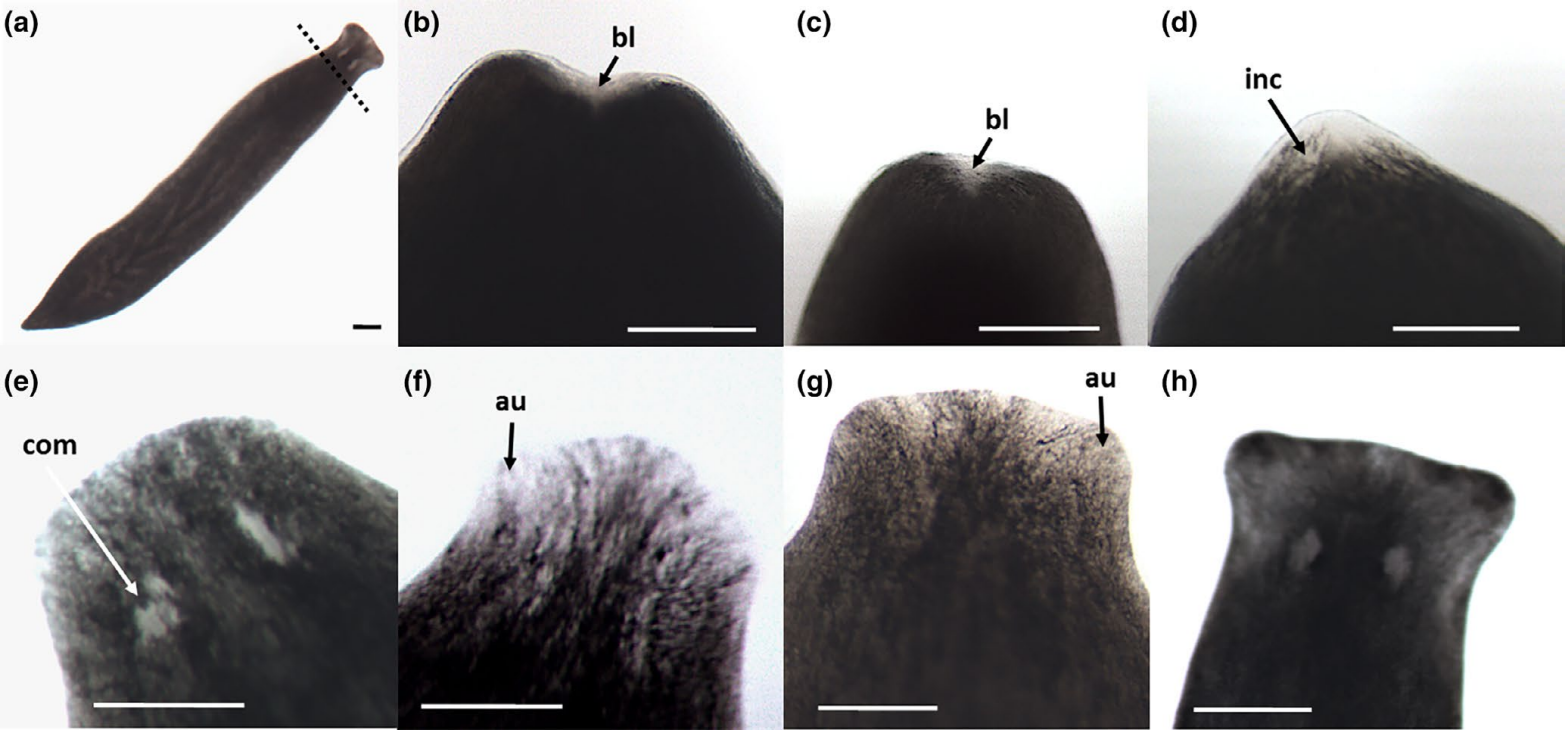
Intact and head regenerating *Phagocata gracilis*. (a) Intact subject. Dashed line indicates level of decapitation; (b) Stage 1; (c) Stage 2; (d) Stage 3; (e) Stage 4; (f) Stage 5; (g) Stage 6; (h) Stage 7; au, auricles; bl, blastema; com, complete eyes; inc, incomplete eyespots; scale bars = 500 μm.

All experiments were initiated by mechanical removal of subjects' heads (decapitation), followed by monitoring head regeneration for 14 days post‐decapitation. Planaria were decapitated in dampened glass Petri dishes by removing all tissue anterior the isthmus that demarcates the head of *P. gracilis* (Figure 1a) using an edge of a plastic microscope slide cover as a blade. Immediately following decapitation, subject bodies were placed individually into uniquely coded 60 mm plastic Petri dishes preloaded with 15 mL of control (carbon‐filtered tap water) or melatonin (0.25 mM aqueous) culture medium. Melatonin (Tocris™, >99% purity) solution was prepared by dissolution in filtered tap water via manual agitation. This concentration is intermediate between concentrations (0.1 and 0.5 mM) previously demonstrated (Beeching & Merritt, 2019) to induce regenerative anomalies dose‐dependently in *P. gracilis* over a 14‐day period.

Following decapitation (Day 0), subjects were held in complete darkness at 22 ± 1°C using a Fisher Isotemp Incubator. Once every 24 h (from Day 1 through Day 14), subjects were observed and scored for stage of regeneration using an Olympus SZ61 stereo microscope. Total time outside of the incubator each day was less than 1 hour for all subjects. Laboratory temperature was always ±2°C of the 22°C incubator temperature. The progressive seven‐stage scoring system (Table 1) of Beeching and Merritt (2019) was employed to assign each subject a daily regeneration score based upon readily identifiable visual endpoints. Regenerative scores range from the initial formation of a blastema (Stage 1, Figure 1b) through complete eye formation (Stage 4, Figure 1e) up to maximum auricle regeneration (Stage 7, Figure 1h). Subjects were randomly assigned to treatment media. In order to guarantee an unbiased analysis, the observers (i.e., each of the co‐authors) had petri dishes assigned an alpha‐numeric code and loaded with culture fluid (water or melatonin solution) by another assisting researcher for each trial. Melatonin is colorless and odorless in aqueous solution. The key to the codes indicating experimental treatment for each subject was kept by the assistant and only revealed during statistical analysis.

**TABLE 1:** Seven stage scoring criteria for head regeneration in *Phagocata gracilis*.

Stage	Criteria
1	Blastema (unpigmented cells) fills 50% or more of invaginated wound site
2	Blastema extends beyond anterior limits of wound site
3	Blastema includes incomplete eyespots without pigmented spot
4	Eyespots are complete with pigmented spot
5	Auricles extend up to 30% of normal, unmanipulated subjects
6	Auricles extend between 30% and 50% of normal, unmanipulated subjects
7	Auricles extend >50% of normal, unmanipulated subjects

### 2.3 Experimental design and data analysis

We first compared the effect of continuous 0.25 mM melatonin exposure (MLT) with continuous water exposure controls (CTL) to model regeneration under each condition. Next, we examined the effects of time‐restricted melatonin exposure using two additional experimental treatments. Weekly exposure experiment trials compared weekly (first or second week only) melatonin‐exposed subjects with simultaneous MLT and CTL subjects. Finally, we conducted 4‐day (Day 4–7) melatonin “pulse” trials, similarly matched with MLT and CTL subjects. Given both the latency to, and recovery from, melatonin effects, we chose a pulse exposure during the late first week that could permit both detection of, and recovery from, melatonin effects during the 14‐day trial, and which coincided with the plateau period effects revealed in our continuous treatment trials. When a protocol required a change in culture medium, all subjects, including MLT and CTL, received same‐day culture medium refreshing.

Daily regeneration scores for each experiment were plotted then analyzed using linear mixed‐effects models (lme4 package for R statistical software, R Core Team, 2021). Specifically, “Treatment”, “Day”, and “Trial” were modeled as fixed effects, with “Subject” as a random effect. For hypothesis testing of treatment × day effects, we used Bonferroni's correction to protect against multiple comparison effects. Treatment effects for both maximum stage achieved and survivorship (days) were tested using Kruskal–Wallis and Dunn's tests. Details of survivorship by day were further explored using the “survive” package for R.

## 3 RESULTS

### 3.1 Continuous melatonin exposure trials

**FIGURE 2:**
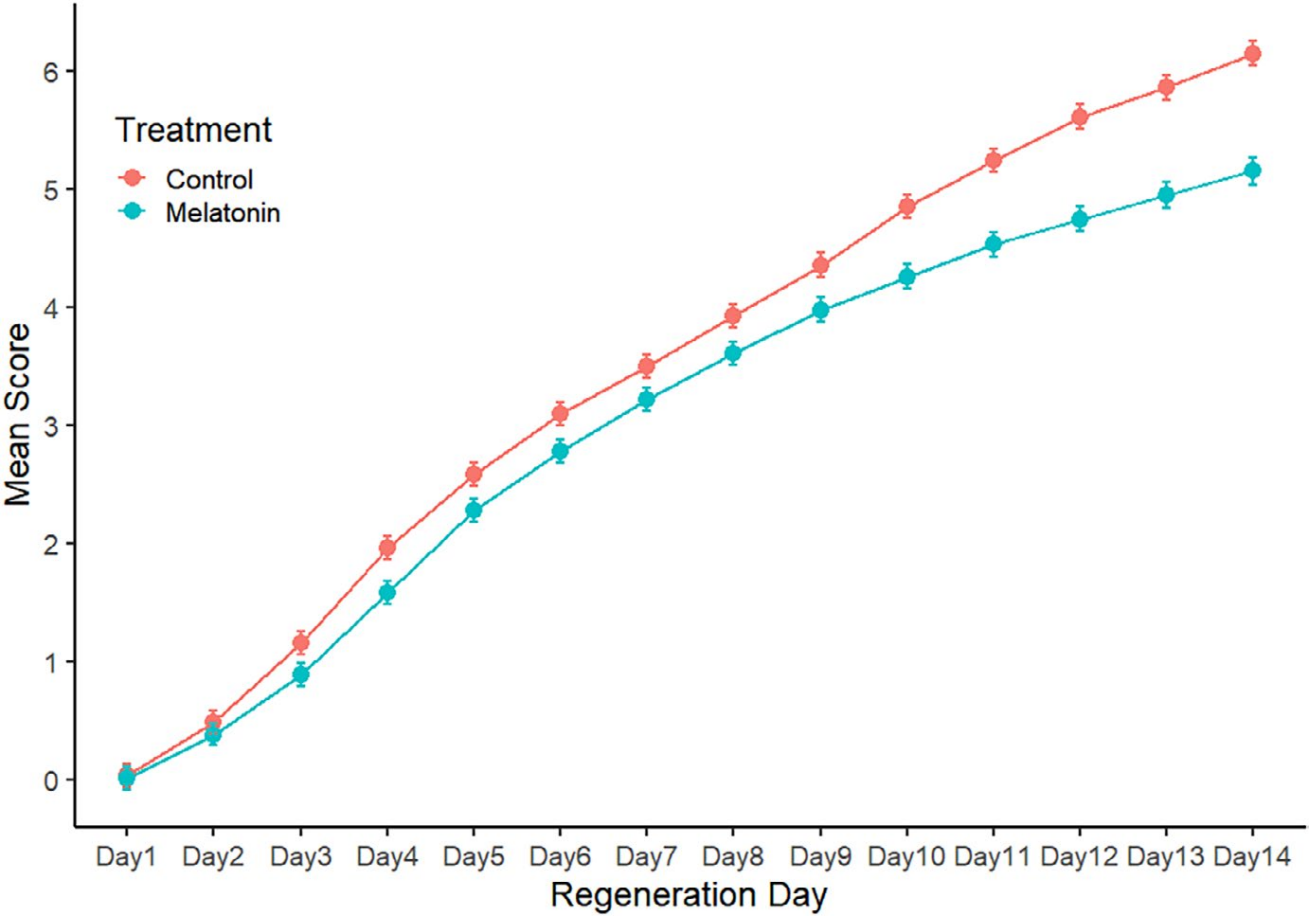
Mean (±95% CIs) daily regeneration scores for control and 0.25 mM melatonin treatment regenerates (initial *n* = 241 for each).

**FIGURE 3:**
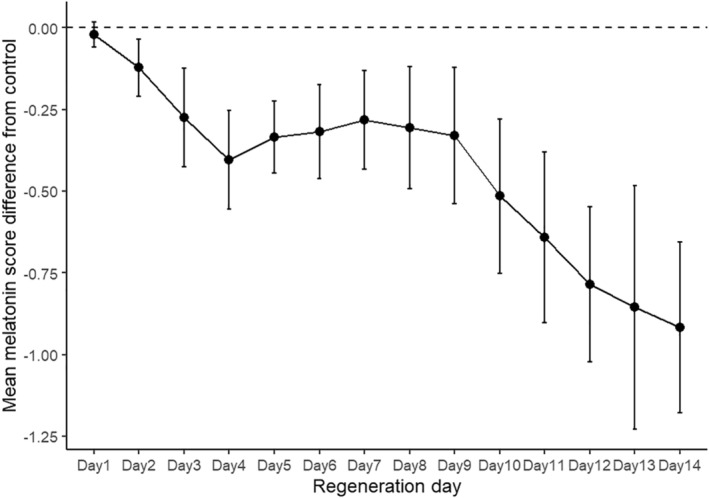
Daily (±95% CIs) mean regeneration scores for melatonin treated regenerates (as Figure 2) plotted as difference from controls.

Regeneration scores for 241 water control (CTL) and 241 continuous 0.25 mM melatonin (MLT) subjects from 22 trials were pooled to determine baseline control and melatonin exposure regeneration parameters. MLT subjects experienced significantly reduced head regeneration scores by Day 3 post‐decapitation, and scores remained significantly lower through Day 14 (Figure 2). Between‐treatment differences generally increase throughout the 14‐day trials and are maximized at Day 14. However, when daily mean melatonin scores were plotted as difference from CTL means (Figure 3), a Day 4 to Day 9 plateau in score differential was revealed.

**FIGURE 4:**
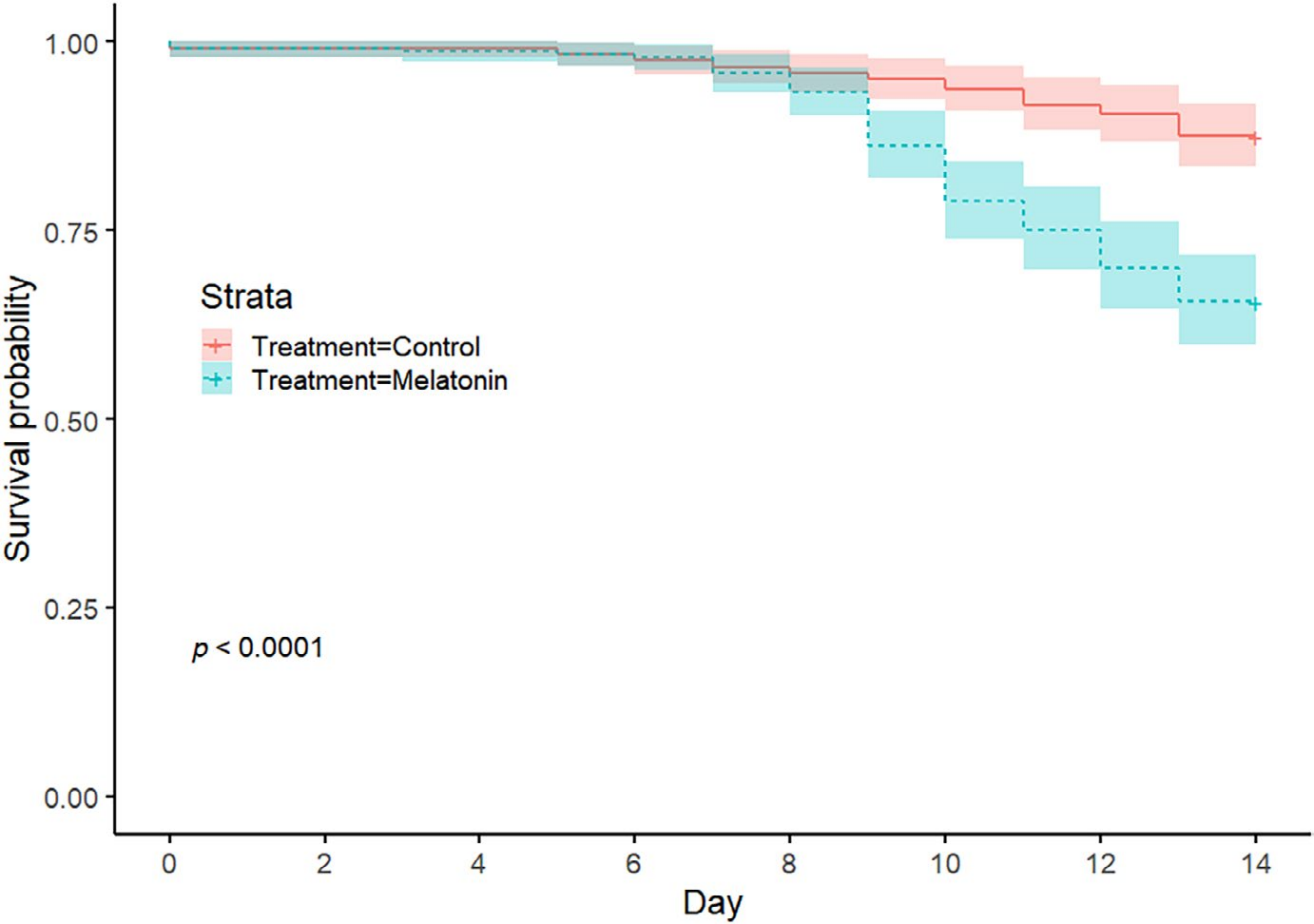
Survivorship probability plots (with 95% CIs) for control and melatonin treated regenerates.

Maximum stage achieved was significantly higher for CTL regenerates ($\bar{x}$ = 5.9) than for MLT treated ($\bar{x}$ = 4.8; Kruskal–Wallis, *X* ^2^ = 104, df = 1, *p* < 0.0001). By Day 14, 97 (40.2%) CTL subjects had reached Stage 7 compared to 21 (8.7%) of MLT subjects. Melatonin treatment reduced survivorship as well (log‐rank test, *X* ^2^ = 32.3, df = 1, *p* < 0.001). Survival probability plots reveal significant between‐treatment differences by Day 9 (Figure 4).

### 3.2 First and second week melatonin exposure trials

**FIGURE 5:**
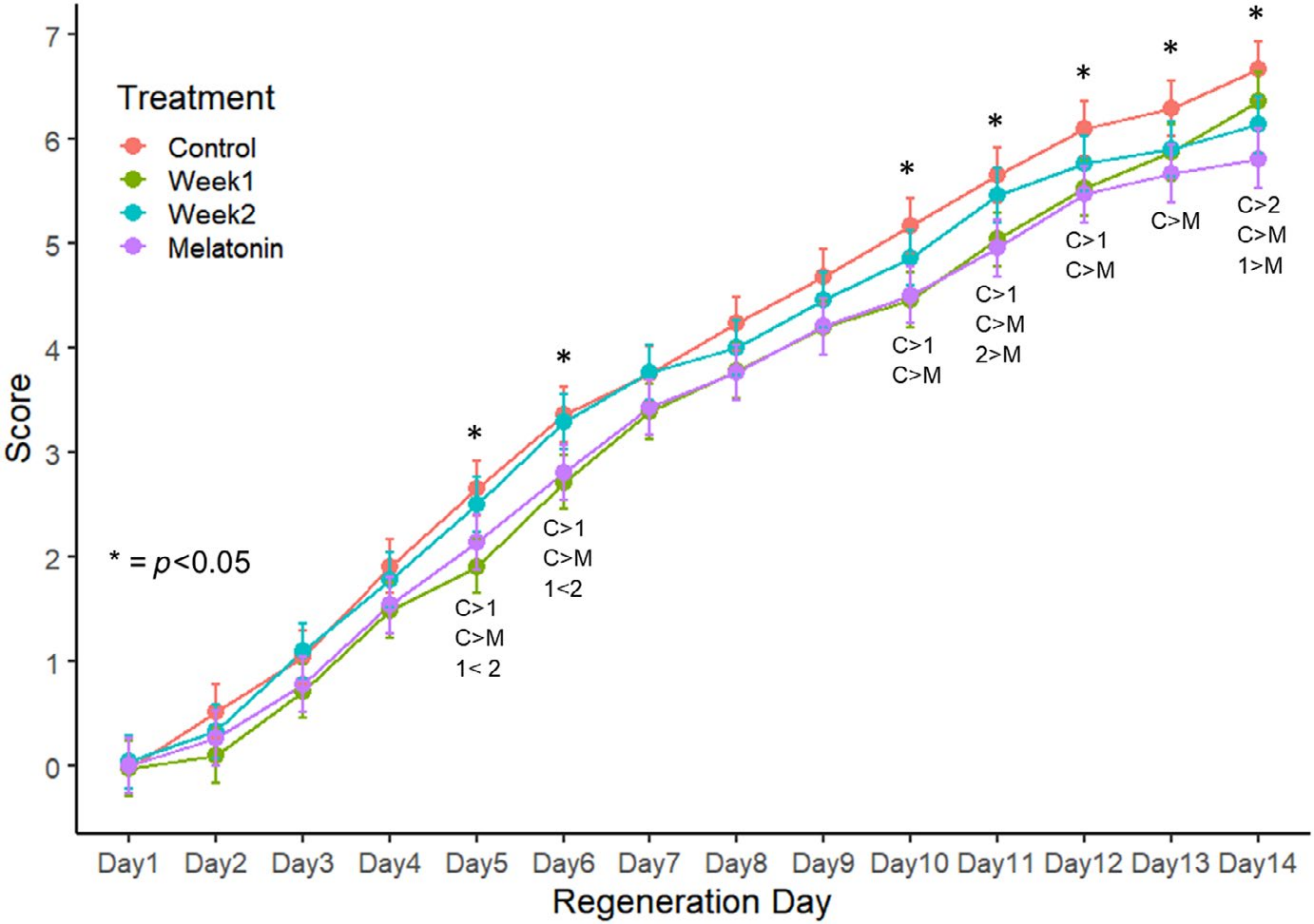
Mean (±95% CIs) daily regeneration scores for water control (C), week 1 (1), week 2 (2), and melatonin (M) treatment regenerates (initial *n* = 31 for each). Days with significant between‐group differences are indicated by an asterisk. Groups and directions of differences are indicated below daily points.

**FIGURE 6:**
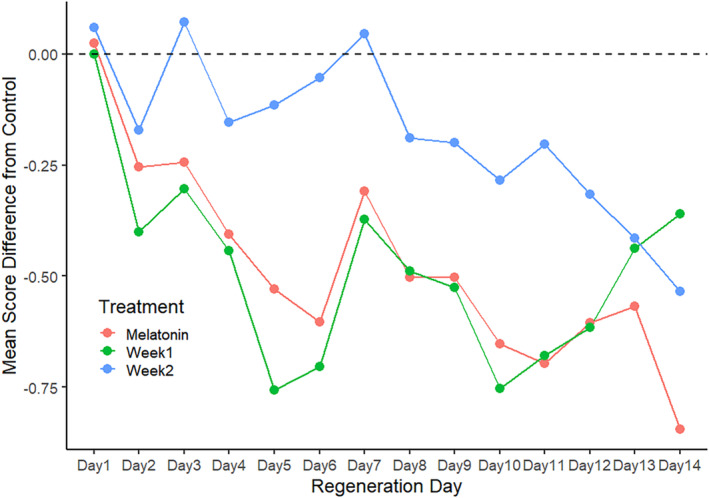
Daily mean regeneration scores for week 1, week 2, and melatonin treatment groups (as Figure 5) plotted as difference from controls.

Four trials comparing weekly melatonin exposure (“Week 1”, “Week 2”) with both CTL and continuous MLT subjects were performed. In total, *n* = 31 subjects were scored for each of the four treatment groups. Both weekly and continuous melatonin exposure affected regeneration scores when compared with controls (Figure 5). Significant differences first appear Day 5, and a convergence of scores was noted from Day 7 through Day 9. Difference from control plots reveal that Week 2 subjects experience inhibitory effects by Day 10. Regeneration in Week 1 subjects paralleled that of MLT subjects until Day 12, but they then recover to near CTL levels by Day 14 (Figure 6).

**FIGURE 7:**
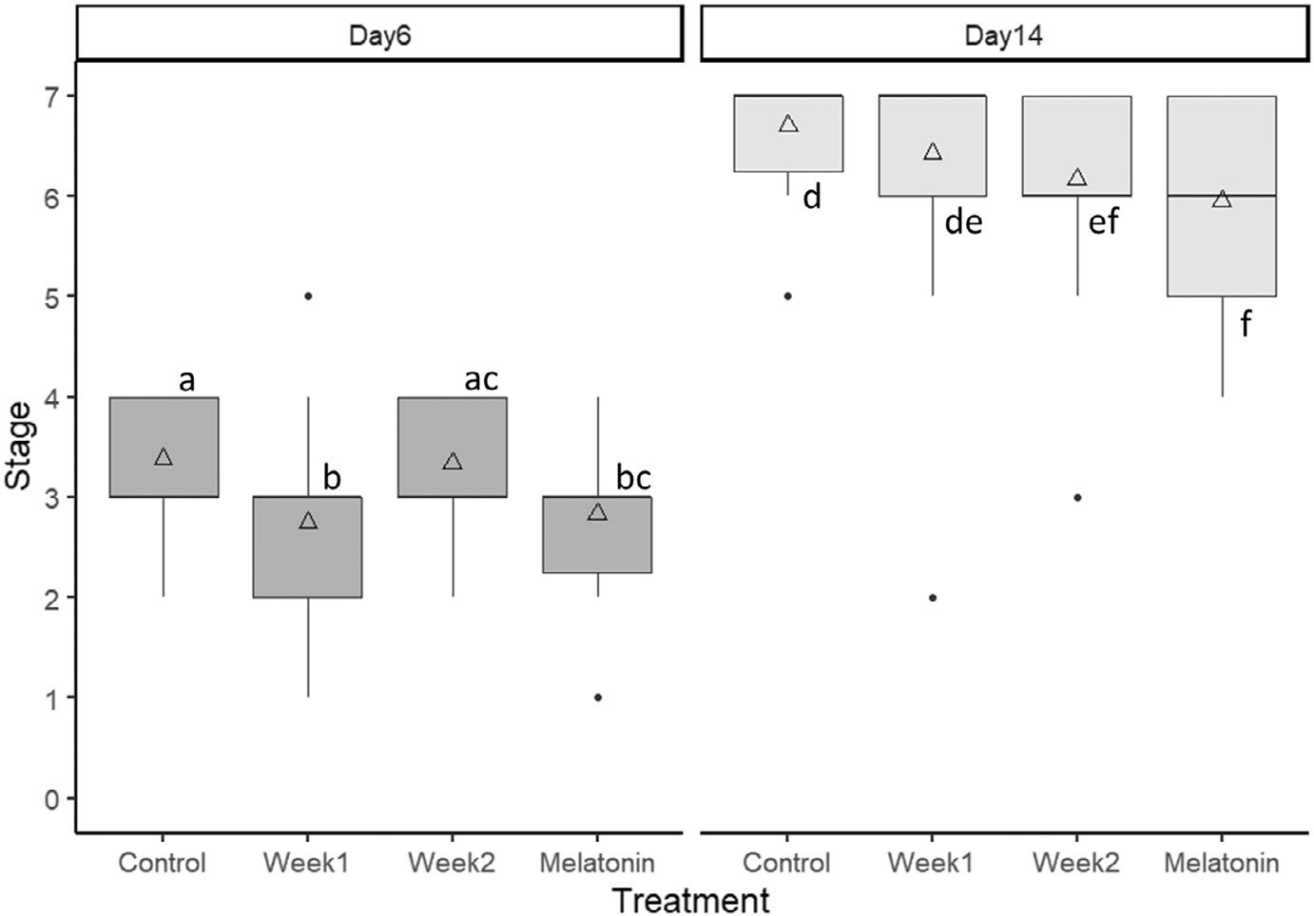
Box plots of scores for control, week 1, week 2, and melatonin groups at Day 6 and 14. Groups sharing the same letter do not differ significantly.

By Day 6, regeneration in both Week 1 and MLT subjects is significantly delayed relative to CTL. By Day 14, Week 1 has recovered to control level, while Week 2 has been delayed to MLT level regeneration (Figure 7). Differences in survivorship among treatments were not significant.

### 3.3 Day 4 through day 7 melatonin pulse trials

**FIGURE 8:**
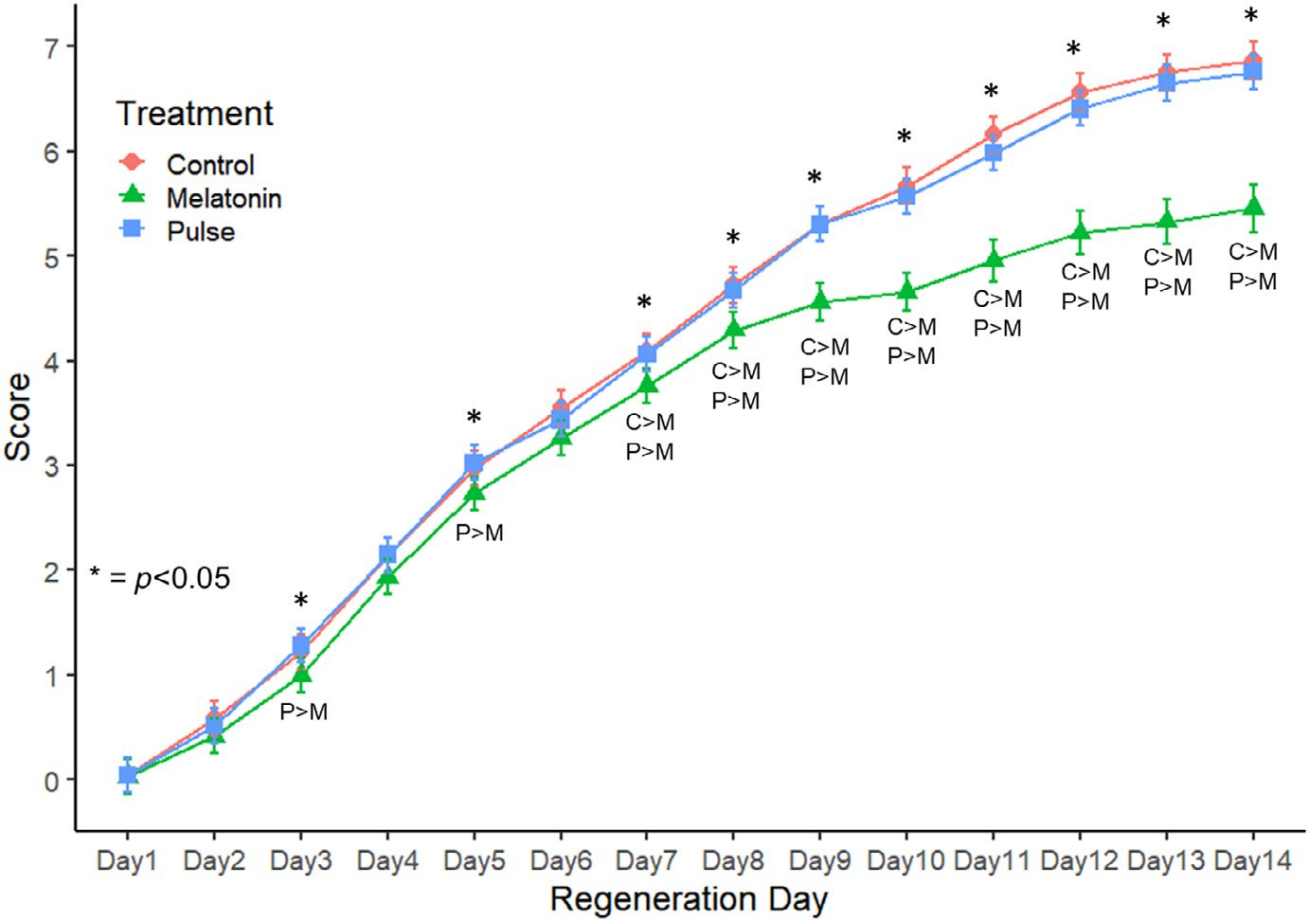
Mean (±95% CIs) daily regeneration scores for water control (C), pulse (P), and melatonin (M) treatment regenerates (initial *n* = 60 for each). Days with significant between‐group differences are indicated by an asterisk. Groups and directions of differences are indicated below daily points.

**FIGURE 9:**
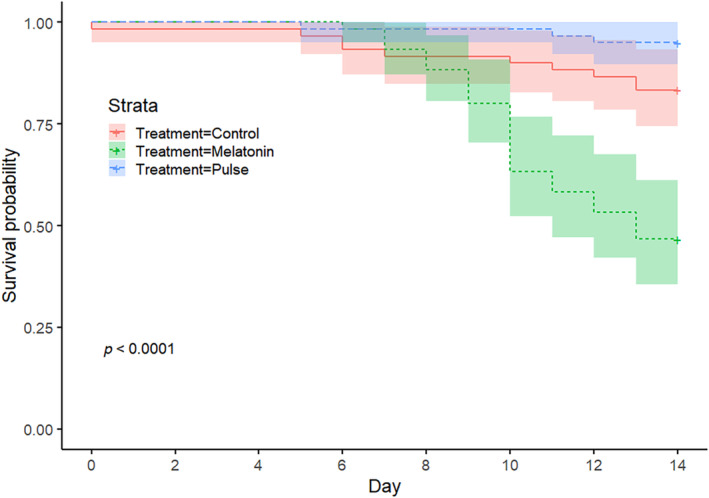
Survivorship probability plots (with 95% CIs) for control, pulse and melatonin treated regenerates.

A total of five trials tested the effect a melatonin pulse from Day 4 to Day 7, with a total of *n* = 60 in each treatment group (i.e., CTL, MLT, and Pulse). Pulse subject daily scores never differed from CTL, while MLT subjects lagged significantly behind both from Day 7 to Day 14 (Figure 8). Both CTL and Pulse regenerates had significantly higher survivorship (Kruskal–Wallis, X^2^ = 38.3, df = 2, *p* < 0.0001) and maximum stage scores (Kruskal–Wallis, *X* ^2^ = 72.8, df = 2, *p* < 0.0001) than MLT subjects. Survival probability analysis revealed that Day 4–7 melatonin pulse treatment did not negatively impact survivorship at any point in the experiment (Figure 9). The time (days) to each stage for continuous, weekly, and pulse trial subjects, along with sample sizes and survivorship, are presented in Table 2.

**TABLE 2:** Mean days to reach Stages 1 through 7, maximum (Max) stage reached, sample size (*n*) and percent survivorship for each experimental treatment group.

Experiment: Exposure	Treatment	Stage							Max	*n*	Survival (%)
		1	2	3	4	5	6	7			
1: Continuous	Control	3	4	6	8	10	14	–	6	241	88
	Melatonin	3	5	7	9	13	–	–	5	241	66
2: Weekly	Control	3	4	6	8	10	11	–	7	31	97
	Week 1	3	5	6	9	11	13	–	6	31	90
	Week 2	3	4	6	8	10	13	–	6	31	94
	Melatonin	3	5	6	9	11	–	–	5	31	77
3: Pulse	Control	3	4	5	7	9	11	–	6	60	83
	Pulse	3	4	5	7	9	11	–	7	60	95
	Melatonin	3	4	6	8	11	–	–	5	60	47

## 4 DISCUSSION

One of the most effective ways to investigate developmental and regenerative processes is to use morphogenic compounds capable of predictably altering embryogenesis, regeneration, and wound healing. Previous studies demonstrated the effectiveness of melatonin in delaying and disrupting planarian regeneration (Beeching & Merritt, 2019; Yermakova et al., 2009; Yoshizawa et al., 1991). Our results confirm the effectiveness of melatonin as a modulator of regeneration in *P. gracilis* and reveal quantitatively different degrees of inhibition well correlated with the stage of regeneration. Under continuous melatonin exposure, *P. gracilis* head regeneration increasingly slowed relative to controls until Day 4, but did not diverge further from controls until Day 10 (Figure 2). This plateau suggests melatonin's effectiveness may be dependent on the specific morphogenic process normally occurring during this five‐day period, most notably eye formation. This finding is consistent with those of Yoshizawa et al. (1991) who noted that melatonin delayed head regeneration but not eye formation in head‐regenerating *Dugesia japonica*. Our control subjects typically reached Stage 2 (blastema extension) on Day 4 and completed eye regeneration (Stage 4) by Day 9. Thus, both earlier (blastema extension) and later (auricle extension) processes exhibit more pronounced inhibition by melatonin.

Melatonin appears to affect other planarian physiological processes in a similarly selective manner. For example, Morita and Best (1984) reported that melatonin suppressed asexual fission in regenerating *Dugesia dorotocephala*, and Yermakova et al. (2009) found melatonin inhibited head, but not tail regeneration in another North American turbellarian, *Girardia tigrina*. More fundamentally, we also confirmed melatonin's dose‐dependent lethal effects on *P. gracilis* previously described by Beeching and Merritt (2019). It remains to be determined if lower concentrations of melatonin exhibit the same inhibitory and lethal properties. However, we were able to examine the effects of lower total melatonin exposure by performing seven‐day (weekly) and four‐day (pulse) and trials.

First week melatonin‐treated subjects experienced delayed head regeneration but recovered to control level following cessation of exposure. Recovery required 6 days and was not statistically complete until Day 13. Second week, melatonin‐treated subjects also experienced delay, but only differed from controls by Day 14. Thus, initiation or cessation of melatonin treatment after Day 7 both produced significant but relatively slow changes in regenerative rate. Here too, regeneration rate stabilized in comparison to controls in melatonin‐treated subjects between Days 5 and 10. Results from both continuous and weekly application of melatonin also suggest a differential response from regenerates during the period of eye formation. Melatonin's nervous tissue‐specific effects have been explored previously in arthropods (Cary et al., 2012; Sainath et al., 2013), and the planarian cerebral ganglion (i.e., “brain”) has been identified as an ideal model for testing neuroregenerative effects of morphogens (Hagstrom et al., 2016). We next examined a melatonin pulse that targeted the mid‐regeneration period of *P. gracilis*.

When subjects were pulsed with melatonin Days 4 through 7, they exhibited a control‐like pattern of regeneration, the second highest survival rate (95%), and the highest mean maximum stage ($\bar{x}$ = 6.7) observed across all trials and treatments. These observations suggest that exogenous melatonin can be applied in ways that both enhance and retard planarian regeneration rate and survivorship. Our results indicate that melatonin's seemingly confounding effects on healing and regeneration are explainable when both melatonin exposure (i.e., concentration and duration) and timing are considered.

Previous reports indicate melatonin can both advance and retard cell proliferation across a variety of taxa and tissues (Agathokleous et al., 2019), and its potential as a therapeutic agent is now widely promoted (Di Bella et al., 2013; Esteban‐Zubero et al., 2016; Liu et al., 2020). Among melatonin's many putative therapeutic functions, its effectiveness as a promoter of tissue regrowth, cell proliferation, and differentiation has been supported by both *in vitro* and *in vivo* studies (Chang et al., 2014; Kandemir & Sarikcioglu, 2015; Luchetti et al., 2014; Stratos et al., 2011). However, some studies have found melatonin to instead inhibit cell proliferation and function in both vertebrates (Letra‐Vilela et al., 2016; Shen et al., 2016) and invertebrates (Sainath et al., 2013). Here too, apparently contradictory results likely reflect biphasic response to melatonin that appears to be typical across an array of taxa (Agathokleous et al., 2019). In considering physiological effects on cancer cells, Bizzarri et al. (2013) concluded that melatonin can be either an inhibitor or promoter of cell death, depending upon variety of factors, including dosage, duration of exposure, and cell type.

Significant questions remain regarding exogenous melatonin's mechanism of action in both vertebrates and invertebrates (Anisimov, 2003), and our results suggest regenerative stage‐specific effects can help elucidate details of melatonin's effects. Melatonin has demonstrated receptor‐mediated effects, including plasma membrane and nuclear receptors (He et al., 2016; Legros et al., 2014), as well as ubiquitous, largely antioxidant receptor‐independent effects (Tan et al., 2013). Beyond its regeneration effects, exogenous melatonin also regulates pigment cell contraction (Csaba et al., 1980) and can induce sleep‐like inactivity (Omond et al., 2017) in planaria. Endogenous melatonin synthesis, including circadian rhythmicity, has also been demonstrated in planaria (Itoh et al., 1999; Morita et al., 1987). Given melatonin's multifaceted role in the biology of planarians, a comprehensive developmental‐endocrine‐physiological approach is required to elucidate its potentially pleiotropic effects on neoblasts during regeneration. The results could be invaluable in exploring the similarly paradoxical effect of exogenous melatonin on cancer cells (Rodríguez‐Santana et al., 2023) and advance the potential of melatonin as a therapeutic agent.

## AUTHOR CONTRIBUTIONS

Simon C. Beeching designed the study. Simon C. Beeching, Hanna E. Ruland, and Katelyn M. Sparks performed experimental manipulations and data collection. Simon C. Beeching performed data analysis, graphic data presentation, photography, and manuscript drafting. Simon C. Beeching, Hanna E. Ruland, and Katelyn M. Sparks collaborated in manuscript editing and revision. All authors approved the final manuscript.

## FUNDING INFORMATION

No funding information provided.

## CONFLICT OF INTEREST STATEMENT

The authors declare no conflict of interest.

## ETHICS STATEMENT

This research complies with the “Guidelines for the Use of Animals in Research” of the Animal Behavior Society and the ARRIVE guidelines of the NC3Rs.

## Data Availability

The data that support the findings of this study are available from the corresponding author upon reasonable request.
